# Malignant Lymphatic and Hematopoietic Neoplasms Mortality in Serbia, 1991–2010: A Joinpoint Regression Analysis

**DOI:** 10.1371/journal.pone.0109379

**Published:** 2014-10-21

**Authors:** Milena Ilic, Irena Ilic

**Affiliations:** Department of Epidemiology, Faculty of Medical Sciences, University of Kragujevac, Kragujevac, Serbia; Kyushu University Faculty of Medical Science, Japan

## Abstract

**Background:**

Limited data on mortality from malignant lymphatic and hematopoietic neoplasms have been published for Serbia.

**Methods:**

The study covered population of Serbia during the 1991–2010 period. Mortality trends were assessed using the joinpoint regression analysis.

**Results:**

Trend for overall death rates from malignant lymphoid and haematopoietic neoplasms significantly decreased: by −2.16% per year from 1991 through 1998, and then significantly increased by +2.20% per year for the 1998–2010 period. The growth during the entire period was on average +0.8% per year (95% CI 0.3 to 1.3). Mortality was higher among males than among females in all age groups. According to the comparability test, mortality trends from malignant lymphoid and haematopoietic neoplasms in men and women were parallel (final selected model failed to reject parallelism, *P* = 0.232). Among younger Serbian population (0–44 years old) in both sexes: trends significantly declined in males for the entire period, while in females 15–44 years of age mortality rates significantly declined only from 2003 onwards. Mortality trend significantly increased in elderly in both genders (by +1.7% in males and +1.5% in females in the 60–69 age group, and +3.8% in males and +3.6% in females in the 70+ age group). According to the comparability test, mortality trend for Hodgkin's lymphoma differed significantly from mortality trends for all other types of malignant lymphoid and haematopoietic neoplasms (*P*<0.05).

**Conclusion:**

Unfavourable mortality trend in Serbia requires targeted intervention for risk factors control, early diagnosis and modern therapy.

## Introduction

Malignant lymphoid and haematopoietic neoplasms (MLHN) are a heterogeneous group of diseases, with different incidence, mortality and survival pattern, with multifactorial etiologies, and without successful prevention or screening strategies [1]–[5]. Based on GLOBOCAN 2008 estimates [6], MLHN cause more than 550,000 deaths per year (accounting for 7.3% of all cancer deaths). Leukemia, with an estimated 257,000 deaths, accounts for 3.4% of all cancer deaths, followed by non-Hodgkin lymphoma (191,599 deaths, 2.5%), multiple myeloma (72453 deaths, 1.0%), and Hodgkin lymphoma (29902 deaths, 0.4%). Almost a half of the deaths from MLHN were registered in Asia in 2008 [6].

During the 1970–2009 period, mortality from leukemia steadily declined in developed countries (in most European countries, the United States of America, Japan) in children and young adults, but decline was not observed at the age of 70 or more [7]. Mortality from non-Hodgkin lymphoma was on the rise until mid-1990s, and started to level off or decline in the following decade in both genders in the European Union, Japan, Australia, however the rates were still increasing in Eastern Europe [8], [9]. Mortality from Hodgkin lymphoma has been decreasing in developed countries in the recent decade (for example, in the United States of America by −2.2% per year for both genders in all races, in Italy by −3.5% per year among men and −3.4% per year among women) [10]–[13]. The highest multiple myeloma mortality rates were reported in developed countries in 2008 [1], [6]. In the European Union, multiple myeloma mortality tended to increase throughout the 1970–2003 period for both genders [14].

The aim of the present study was to assess time trends of mortality from MLHN during the 1991–2010 period in Serbia.

## Materials and Methods

### Data sources

Data on individuals who died from MLHN (codes 200–208 revision 9 of the International Classification of Diseases from 1991 to 1996, and codes C 81–96 revision 10 from 1997) were obtained from Statistical Office of the Republic of Serbia (unpublished data). MLHN were defined as revision 9 of the International Classification of Diseases to classify death, injury and cause of death codes 201 (Hodgkin's lymphoma), 200 and 202 (non-Hodgkin's lymphoma), 203 (Multiple myeloma and malignant plasma cell neoplasms), 204–208 (Leukemia), 204 (Lymphoid leukemia), 205 (Myeloid leukemia), and revision 10 of the International Classification of Diseases to classify death, injury and cause of death codes C81 (Hodgkin's lymphoma), C82-85 and C96 (non-Hodgkin's lymphoma), C88 and C90 (Multiple myeloma), C91-95 (Leukemia), C91 (Lymphoid leukemia), C92 (Myeloid leukemia).

The research included the entire population of the Republic of Serbia, during the 1991–2010 period, excluding the Autonomous Province of Kosovo and Metohia due to unavailability of data since 1998. For the entire study period, data both on people who died of MLHN and on whole population of Serbia were obtained from the Statistical Office of the Republic of Serbia. Data on the number and composition of the Serbian population by gender and age were obtained from the population censuses performed in 1991 and 2002. For inter-census years, the estimates published by the Statistical Office of the Republic of Serbia were used. The analysis was conducted on the entire Serbian population (approximately 7.5 million persons, from about 7.6 million inhabitans in 1991 to about 7.3 inhabitans in 2010) [15]. Data for internally displaced persons and refugees were included in the Serbian population (nearly 400,000 persons), but could not be set aside as a special contingent.

Compliance with international recommendations, standards and practices in Serbia is achieved by conducting research on the dead according to the methodology that is in line with the recommendations of the UN - Principles and Recommendations for a Vital Statistics System Revision 2. All deaths occurring in Serbia are registered in death files (certificate of death and a special statistical form – DEM 2) [16]. A death certificate is obtained from an authorized physician in a health care organization, a coroner, or a forensic physician. Death files are checked by the local registrar and forwarded to the referral public health institute where they are checked again and if necessary corrected by another trained medical doctor or specialist.

### Ethics

This study is part of a larger research approved by the Ethics Committee of the Clinical Center of Kragujevac (protocol: 01-1836).

### Statistical analysis

Three types of death rates were calculated: crude, age-specific and age-standardized. The age standardized rate (ASR) is calculated by the direct method from the World standard population proposed by Segi [17]. Rates are expressed as deaths per 100,000 persons, and are presented by age, gender and type. Age-specific rates are computed for the following age groups: 0–14, 15–44, 45–59, 60–69, 70+ years. The results are not shown for some subgroups, because fewer than 5 cases of MLHN deaths occurred in each of the quinquennium in any year.

Mortality trends from MLHN were assessed using joinpoint regression analysis (Joinpoint regression software, Version 4.0.4 – May 2013, available through the Surveillance Research Program of the US National Cancer Institute). The joinpoint analysis was applied to assess the mortality trends for MLHN, detect when statistically significant changes in the trend occur, and determine the trends between joinpoints. The joinpoint regression analysis includes a series of joined straight lines on a log scale to the trends in the annual age-standardized mortality rates. The tests of significance use the Monte Carlo Permutation method [18]. Each joinpoint denotes a statistically significant (*P* = 0.05) change in a trend. Changes in annual mortality rates from MLHN were calculated as the annual percentage change (APC) in each segment, and estimates of annual average of APC with corresponding 95% confidence intervals were made [19]. In the final model, the joinpoint analysis provided average annual percentage change (AAPC); for each AAPC estimate, researchers calculated the corresponding 95% CI. Disparities in mortality trends according to age, gender and type were tested by using a comparability test – a procedure proposed by Kim et al. [20]. The goal of the comparability test was to answer whether the two regression mean functions were identical (test of coincidence) or parallel (test of parallelism).

## Results

**Figure 1:**
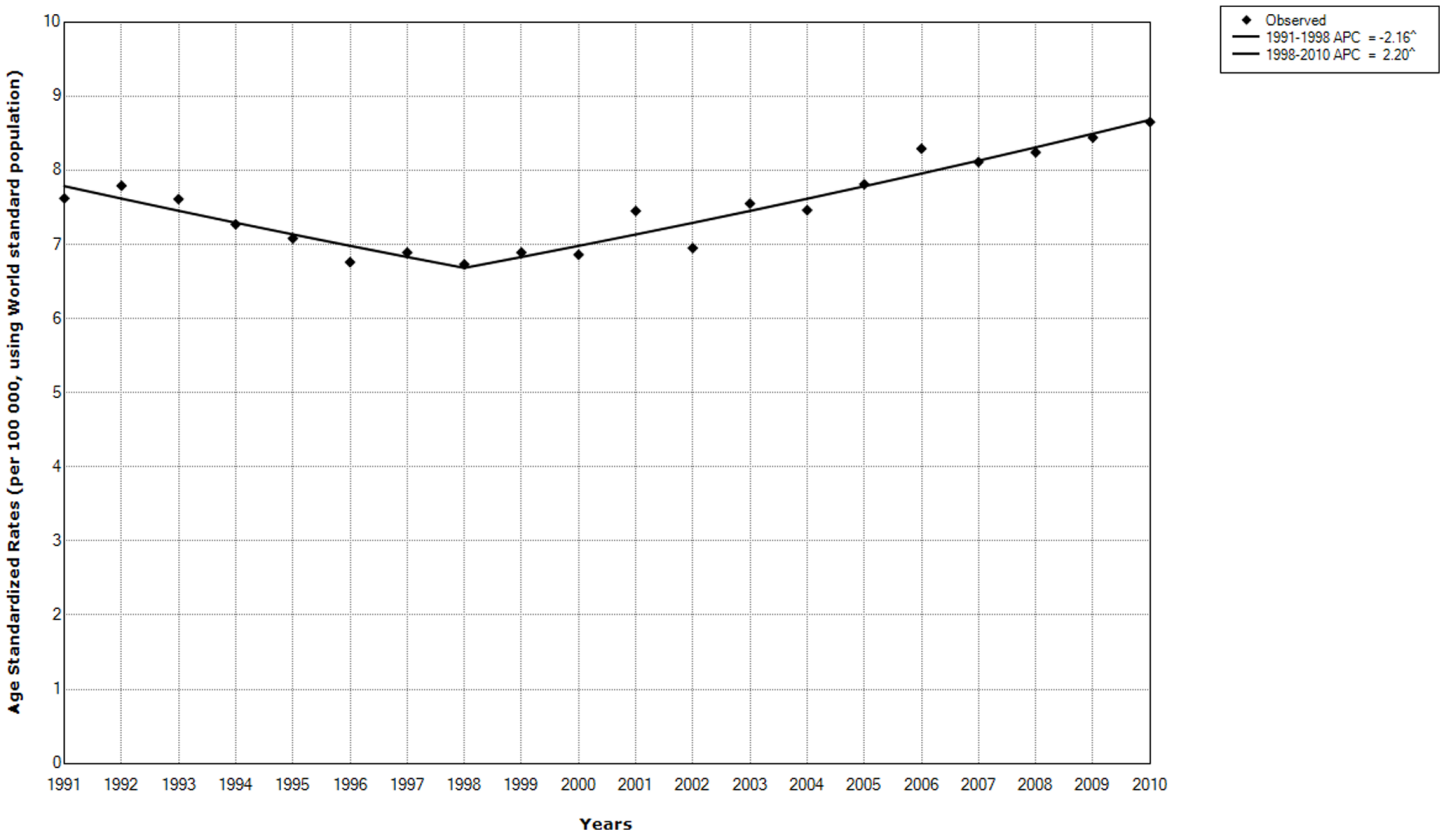
Malignant lymphatic and hematopoietic neoplasms (according to ICD-10: codes C 81–96). Age-standardized mortality rates, per 100 000 inhabitants, using World standard population (marked with diamonds). Based on the results of joinpoint regression analysis, one corresponding joinpoint and two trends (marked with two lines) had been identified for the overall mortality trend in Serbia 1991–2010. Annual Percentage Changes (APC) are given. * *p*<0.05.

The joinpoint trend for overall death rates from MLHN significantly decreased by −2.16% per year from 1991 through 1998, then increased by +2.20% per year for the 1998–2010 period in Serbia, excluding the Autonomous Province of Kosovo and Metohia (Figure 1). The overall increase during the whole period was +0.8% on average per year (95% CI 0.3 to 1.3).

During the 1991–2010 period, nearly 18,500 inhabitants (approximately 10,500 men and 8,000 women) died from MLHN (Table 1).

**Table 1:** Malignant lymphatic and hematopoietic neoplasms mortality in Serbia, excluding the Autonomous Province of Kosovo and Metohia, in the period 1991–2010, by gender: Number of cases, crude rate, and age standardized rate.

	Men			Women		
Year	N	Crude rate	ASR	N	Crude rate	ASR
1991	453	12.19	9.36	345	8.89	5.88
1992	484	13.02	9.80	348	8.95	5.77
1993	487	12.95	9.38	343	8.81	5.84
1994	451	12.11	8.84	332	8.52	5.70
1995	439	11.78	8.62	325	8.33	5.53
1996	436	11.71	8.05	340	8.72	5.47
1997	453	12.22	8.36	360	9.25	5.41
1998	435	11.80	8.04	365	9.40	5.42
1999	450	12.26	8.14	371	9.58	5.63
2000	463	12.66	8.14	357	9.25	5.58
2001	502	13.76	8.82	417	10.82	6.07
2002	494	13.54	8.50	376	9.76	5.40
2003	522	14.35	9.23	428	11.14	5.87
2004	532	14.66	9.04	415	10.82	5.87
2005	573	15.84	9.82	426	11.14	5.80
2006	579	16.07	9.65	496	13.03	6.93
2007	638	17.78	10.48	439	11.58	5.73
2008	629	17.60	10.10	491	13.00	6.37
2009	616	17.30	9.85	529	14.07	7.02
2010	681	19.20	10.61	522	13.94	6.68
Overall	10317	14.14	9.14	8025	10.45	5.90

ASR – Age Standardized Rate (per 100 000, using World standard population).

Mortality from MLHN was continuously increasing since 1991 by 0.8% (95% confidence interval [CI] 0.1 to 1.4) yearly in men, and by 0.8% (95% CI 0.3 to 1.4) yearly in women (Table 2). According to the comparability test, mortality trends from MLHN in men and women were parallel (final selected model failed to reject parallelism, *P* = 0.232). There were some gender differences between time trends by age. The trend analysis of MLHN shows a favourable pattern among younger Serbian population (0–44 years old) in both genders: trends declined significantly in males during the entire period, while in females aged 15–44 years mortality rates significantly declined only from 2003 onwards (by −4.3% per year, 95% CI −7.7 to −0.8). Overall, in the 60–69 age group ASRs of mortality significantly increased in both genders (by +1.7% in males and +1.5% in females), but there was a joinpoint in 1997 for males when the significantly decreased trend (by −3.5% per year) continued with a significantly increased trend (+3.5% per year) onwards. In the elderly (population 70+ years) during the entire period ASRs of mortality significantly increased by +3.8% per year for males and +3.6% per year for females; however, for female mortality, there was one joinpoint at 1993: ASRs decreased by −16.8% per year from 1991 to 1993, and then rapidly increased by +4.6% per year from 1993 to 2010.

**Table 2:** Joinpoint regression analysis of malignant lymphatic and hematopoietic neoplasms mortality in Serbia, excluding the Autonomous Province of Kosovo and Metohia, by sex and age, in 1991–2010 period.

Age	Average age-specific rates (per 100 000)	AAPC†	95%CI‡
Male			
0–14	1.65	−4.6*	−6.4 to −2.7
15–44	4.04	−1.6*	−2.7 to −0.4
45–59	15.19	+0.8§	−0.3 to 1.9
60–69	37.47	+1.7* ^,^ **	0.8 to 2.6
70+	55.20	+3.8*	2.8 to 4.9
All males		+0.8* ^,^ ††	0.1–1.4
Female			
0–14	0.93	−7.0*	−11.0 to −2.7
15–44	2.84	−0.8‡‡	−1.8 to 0.3
45–59	10.03	+0.7	−0.1 to 1.6
60–69	23.69	+1.5*	0.6 to 2.4
70+	34.19	+3.6* ^,^ §§	2.4 to 4.8
All females		+0.8* ^,^ †‡	0.3–1.4

* Joinpoint is significantly different from zero at alpha = 0.05;

† Average Annual Percent Change;

‡ CI − Confidence Interval.

§ Two joinpoints, for trend in 45–59 years old males: Trend 1 (1991–1993): annual percent change (APC) (95% CI) = +17.0 (−6.3 to 46.1); Trend 2 (1993–1998): APC (95% CI) = −7.2* (−13.5 to −0.4); Trend 3 (1998–2010): (APC) (95% CI) = +3.2* (1.9 to 4.6).

** One joinpoint, for trend in 60–69 years old males: Trend 1 (1991–1997): (APC) (95% CI) = −3.5* (−6.7 to −0.2); Trend 2 (1997–2010): APC (95% CI) = +3.5* (2.5 to 4.6).

†† One joinpoint, for trend in all males: Trend 1 (1991–1998): (APC) (95% CI) = −2.7* (−4.0 to −1.4); Trend 2 (1998–2010): APC (95% CI) = +2.4* (1.8 to 3.1).

‡‡ Three joinpoints, for trend in 15–44 years old females: Trend 1 (1991–1994): annual percent change (APC) (95% CI) = +4.8 (−8.5 to 20.0); Trend 2 (1994–1999): APC (95% CI) = −8.1 (−15.6 to 0.1); Trend 3 (1999–2003): (APC) (95% CI) = +10.1 (−3.8 to 26.0); Trend 4 (2003–2010): (APC) (95% CI) = −4.3* (−7.7 to −0.8).

§§ One joinpoint, for trend in 70+ years old females: Trend 1 (1991–1993): (APC) (95% CI) = −16.8 (−40.3 to 16.0); Trend 2 (1993–2010): APC (95% CI) = +4.6* (3.4 to 5.8).

†‡ One joinpoint, for trend in all females: Trend 1 (1991–1998): (APC) (95% CI) = −1.3 (−3.3 to 0.8); Trend 2 (1998–2010): APC (95% CI) = +1.8* (0.9 to 2.8).

The mortality by particular type of MLHN is presented in Table 3. Hodgkin's lymphoma's mortality rates significantly decreased by −1.4% per year (95%CI −2.5 to −0.2) over the entire period. There were three joinpoints: the rate increased by +19.7% (95% CI −2.1 to 46.4) per year from 1991 to 1993, and then significantly decreased by −9.3% (95% CI −13.3 to −5.1) per year from 1993 to 1999. Afterwards, the trend increased by +12.2% (95% CI −8.2 to 37.2) per year from 1999 to 2002, than significantly decreased by −3.4% (95% CI −5.5 to −1.2) from 2002 onwards. Since 1991, non-Hodgkin's lymphoma mortality rates have significantly increased among Serbian population by +2.7% per year (95% CI 1.9 to 3.5). Multiple myeloma's mortality significantly increased by +2.5% (95%CI 1.8 to 3.3) per year during the entire period. Leukemia's death rates were stable throughout the period (AAPC = +0.2%, 95% CI −0.4 to 0.8). Lymphoid leukemia's death rates non-significantly declined throughout the period, AAPC was −0.4% (95%CI −1.1 to 0.2). Myeloid leukemia's mortality rates were significantly increased by +1.7% (95% CI 0.5 to 2.9) per year.

**Table 3:** Joinpoint regression analysis* (of age-specific and age-standardized rates) of malignant lymphatic and hematopoietic neoplasms mortality rates per 100,000 persons in Serbia, excluding the Autonomous Province of Kosovo and Metohia, by type and age, in 1991–2010 period.

1991–2010	HL	NHL	MM	Leukemia	LL	ML
Total number	1491	4649	2724	9478	4164	3645
ASR† (averal annual)	0.69	1.84	0.97	3.85	1.67	1.52
AAPC‡ (95%CI§)	−1.4* ^,^ †† (−2.5 to −0.2)	+2.7* (1.9 to 3.5)	+2.5* (1.8 to 3.3)	+0.2‡‡ (−0.4 to 0.8)	−0.4 (−1.1 to 0.2)	+1.7* ^,^ §§ (0.5 to 2.9)
**Age** (years)						
0–14						
Average age-specific rates	0.05	0.22	0.00	0.20	0.62	0.15
AAPC (95%CI)**	–	–	–	–	−2.3 (−5.6 to 1.1)	–
15–44						
Average age-specific rates	0.66	0.85	0.10	0.21	0.67	0.91
AAPC (95%CI)**	+0.5 (−1.4 to 2.3)	0.0 (−1.7 to 1.7)	–	–	−0.6 (−3.0 to 1.9)	−1.7* (−3.0 to −0.5)
45–59						
Average age-specific rates	1.24	3.44	1.92	0.94	1.94	3.07
AAPC (95%CI)	−3.2* (−5.1 to −1.3)	+3.0* (2.0 to 4.1)	+2.3* (0.8 to 3.7)	−4.2* (−6.8 to −1.6)	−2.2* (−3.7 to −0.6)	+2.0* (0.2 to 3.8)
60–69						
Average age-specific rates	1.83	7.77	5.71	2.72	6.28	5.56
AAPC (95%CI)	−3.7* (−6.1 to −1.2)	+4.1* (2.7 to 5.5)	+2.5* (1.3 to 3.8)	−0.3 (−2.0 to 1.5)	−0.7 (−1.7 to 0.4)	+2.6* ^,^ §† (0.9 to 4.2)
70+						
Average age-specific rates	2.10	9.73	5.82	4.93	12.99	6.32
AAPC (95%CI)	+1.0 (−1.2 to 3.3)	+4.9* ^,^ †‡ (3.1 to 6.8)	+4.6* (2.6 to 6.7)	+1.6†§ (−0.2 to 3.4)	+2.7* ^,^ ‡§ (1.5 to 3.8)	+5.8* (3.7 to 8.0)

* Joinpoint is significantly different from zero at alpha = 0.05;

† ASR – Age Standardized Rate (per 100 000, using World standard population);

‡ Average Annual Percent Change;

§ CI − Confidence Interval;

** Joinpoint results are not shown for this age subgroup, since there were less than 5 cases in any given year.

Abbreviations: HL (Hodgkin's lymphoma), NHL (Non-Hodgkin's lymphoma), MM (Multiple myeloma), LL (Lymphoid leukemia), ML (Myeloid leukemia).

†† Three joinpoints, for HL trend in overall: Trend 1 (1991–1993): annual percent change (APC) (95% CI) = +19.7 (−2.1 to 46.4); Trend 2 (1993–1999): APC (95% CI) = −9.3* (−13.3 to −5.1); Trend 3 (1999–2002): (APC) (95% CI) = +12.2 (−8.2 to 37.2); Trend 4 (2002–2010): (APC) (95% CI) = −3.4* (−5.5 to −1.2).

‡‡ One joinpoint, for leukemia trend in overall: Trend 1 (1991–1998): (APC) (95% CI) = −2.2 (−4.4 to 0.1); Trend 2 (1998–2010): APC (95% CI) = +1.3* (0.3 to 2.3).

§§ One joinpoint, for ML trend in overall: Trend 1 (1991–1999): (APC) (95% CI) = −3.1* (−6.0 to −0.1); Trend 2 (1999–2010): APC (95% CI) = +4.9* (2.9 to 6.9).

†‡ One joinpoint, for NHL trend in 70+ years old persons: Trend 1 (1991–1995): (APC) (95% CI) = −9.7 (−22.5 to 5.2); Trend 2 (1995–2010): APC (95% CI) = +7.3* (5.1 to 9.5).

†§ Two joinpoints, for all leukemia trend in 70+ years old persons: Trend 1 (1991–1993): annual percent change (APC) (95% CI) = −29.9 (−57.0 to 14.5); Trend 2 (1993–1996): APC (95% CI) = +25.9 (−22.9 to 105.6); Trend 3 (1996–2010): (APC) (95% CI) = −0.3 (−2.6 to 2.0).

‡§ One joinpoint, for LL trend in 70+ years old persons: Trend 1 (1991–1996): (APC) (95% CI) = −4.5 (−11.1 to 2.6); Trend 2 (1996–2010): APC (95% CI) = +4.3* (2.8 to 5.9).

§† One joinpoint, for ML trend in 60–69 years old persons: Trend 1 (1991–2000): (APC) (95% CI) = −3.0 (−6.0 to 0.2); Trend 2 (2000–2010): APC (95% CI) = +7.6* (4.6 to 10.6).

The age-specific mortality rates for all types of MLHN grew with age (Table 3). Statistically significant increased mortality trends for non-Hodgkin's lymphoma, multiple myeloma, lymphoid leukemia and myeloid leukemia were observed in the entire study period among persons aged 70 years and over, while increased mortality trends for Hodgkin's lymphoma and all leukemia were not significant. Death rates from non-Hodgkin's lymphoma, multiple myeloma and myeloid leukemia were significantly increased in persons 45–69 years old, but the decreasing mortality trends for Hodgkin's lymphoma and lymphoid leukemia are encouraging. Statistically significant downward trend for mortality from myeloid leukemia was recorded in the age group 15–44 years.

## Discussion

This study describes temporal trends of mortality from MLHN in Serbia in the last two decades. The main finding of this study is the significant increase of overall mortality from MLHN in Serbia. A rise in mortality rates started in 1998, and was identified in both genders. The increasing trend was more pronounced in the elderly. Significant decrease of mortality in younger age groups in both genders could be promising. The overall increase in mortality is mostly attributable to rise in non-Hodgkin's lymphoma, multiple myeloma and myeloid leukemia.

Most of the developed countries showed a similar increase in mortality from non-Hodgkin's lymphoma in both genders since 1960 (United States of America, United Kingdom, Netherlands, Switzerland, Norway, Sweden, Japan, France, Finland, Canada, Australia), but the rates decreased from 1995 onwards [6], [10]. Also, data for the 27 European Union member states showed that mortality from non-Hodgkin's lymphoma peaked in the late 1990s and declined thereafter in both genders (by −1.3% per year in men and −2.1% per year in women) [14]. The rates were, however, still increasing in eastern European countries (in the Russian Federation in both genders, in women in Romania, Latvia and Slovakia) [8]. Serbia is among the countries with the lowest mortality rates from non-Hodgkin's lymphoma in the world, but mortality continually increased by +2.7% per year (95% CI 1.9 to 3.5%) during the 1990–2010 period.

The temporal trends in mortality from non-Hodgkin's lymphoma, in addition to changes in incidence or classification of disease, likely reflect improvements of treatment and survival. The incidence of non-Hodgkin's lymphoma has been rising in many countries during the last few decades (United States, Australia, Nordic countries, Croatia, Uganda) [9], [10], [21]–[24]. Although the etiology of non-Hodgkin's lymphoma remains largely unknown, the incidence increase probably predominantly reflects trends in the incidence of HIV [25], and then the spread of hepatitis C virus and other infectious agents [26], the increased number of transplantations, exposure to radiation, drugs, and occupational exposure to chemicals [27], obesity [28], diabetes mellitus [29], tobacco smoking [30], dietary habits [31], drinking of alcohol [32]. The decrease in mortality from non-Hodgkin's lymphoma in developed countries was attributed to the declining incidence of HIV infection and the introduction of highly active antiretroviral therapies since 1996 [33]. The recent significant increasing trends of non-Hodgkin's lymphoma survival in the United States [10] and the Nordic countries [23], and considerably lesser survival rates in Eastern Europe [34] are likely to be the important reason for intercountry differences in non-Hodgkin's lymphoma mortality.

Given the lack of national incidence and survival rates for non-Hodgkin's lymphoma in Serbia, the available data do not allow complete explanation of this unfavorable mortality trend in Serbia. In Serbia, the turn of the century was marked by wars, international economic embargo and all the negative consequences they produce, such as the collapse of all segments of society, and therefore the health care system. The hospitalisation rates declined significantly in 1990s, particularly for people aged 60 years and older, while at the same time mortality of hospitalised patients has increased, particularly for the elderly, probably as a result of the inability to import needed medications [35], [36]. According to the data of one Belgrade hospital collected for the 2000–2007 period, the treatment response and survival rate in the elderly non-Hodgkin's lymphoma patients were significantly poorer than in younger patients, the fact explained by presence of comorbidities and treatment toxicity [37]. The increased trend in incidence and mortality of HIV in Serbia was registered in the 1985–1997 period, and has been on the decline since, which is likely the result of the implementation of highly active, antiretroviral therapy - HAART since 1997 [38]. Improvements in diagnosis and certification accuracy may at least partially contribute to the increased trend in non-Hodgkin's lymphoma mortality in Serbia [39], [40]. Although refugee status for the deceased from non-Hodgkin's lymphoma is unknown, the mortality trends in Serbian population correspond to those observed in the neighboring countries [21] that were source of a large number of refugees who fled to Serbia.

All developed countries showed a similar decreasing trend in mortality from Hodgkin's lymphoma [10], [14]. In the 10 European Union accession countries from Central and Eastern Europe, characterised by unfavourable trends up to the 1990s, Hodgkin's lymphoma mortality has started to decline in recent years [11]. Similarly, death rates have been declining in Serbia over the 1991–2010 period, but Hodgkin's lymphoma mortality trend showed no consistent pattern. Although mortality from the Hodgkin's lymphoma is declining in most countries, the incidence is generally reported without a consistent trend (declining, increasing or stable): rates for new Hodgkin's lymphoma cases have been stable over the last 10 years in white and Hispanic, but significantly increased in black and at Asian/Pacific Islander populations in the USA[10], [22]. In developed countries, the favorable patterns for Hodgkin's lymphoma death rates in more recent years are being linked with advancements in treatment with a consequently better survival [10], [23]. In one university hospital in Serbia, the overall survival rate was 76% [41]. The observed patterns of the increased rates of deaths during the 1991–1993 and 1999–2002 periods might have been the consequence of decresead availability of advanced treatment modalities in Serbia in those years [37]. The Yugoslav wars, the United Nations Security Council economic embargo against Serbia and Montenegro between 1992 and 1995, the NATO bombing of Yugoslavia in 1999, which resulted in deterioration of health care in Serbia, had a distinctly adverse effect on mortality from malignant neoplasms.

In a survey of cancer mortality in the European Union, Bosetti et al [14] observed steady increase in mortality from multiple myeloma until 1990s, but with tendency to level off in recent years. In the recent decline in mortality from multiple myeloma, improved treatment may have an important role [42], [43]. Serbia (with rate 1.1 per 100,000) was among the countries with intermediate rates, wherein mortality continuously increased since 1991, by +2.5% per year. The suspected risk factors for multiple myeloma include ionizing radiation, occupational exposures, chemicals, obesity, alcohol, and tobacco [44]–[46]. The multiple myeloma percentage (7.79%) in the group of patients belonging to clean-up workers in study in Chernobyl in the 1996–2005 period turned out to be twice as much as in patients in general population (4.0%) [44]. In a case-control study in Belgrade, it was found that factors associated with the occurrence of multiple myeloma were smoking and rheumatoid arthritis in personal history, while consumption of vegetables had a protective role [45]. According to the National Health Survey data for 2006, 3.4% of the adult population of Serbia drank alcohol on a daily basis, which is an increase of 0.1% in comparison with 2000 (3.3%) [47]. Serbia has joined World Health Organization Convention on Tobacco Control in 2006, and real effect on reduction in the tobacco smoking is expected in the near future.

Mortality from leukemia steadily declined in most European countries, the United States and Japan, in both genders aged 0–69, over the 1970–2009 period [7]. Survival from leukaemia is very poor in the elderly [10], [23]. The favorable trends for mortality from leukemia are associated with diagnosis and/or treatment improvements and better survival in developed countries [23], [48]. However, deaths rates for leukemia have increased in Serbia since 1998. Our data indicated that significant increase in leukemia mortality coincides with the steeply increased mortality rates for myeloid leukemia since 1999. Data about leukemia incidence and survival rate in Serbia are very scanty. There is no single known cause for leukemia. The risk factors for leukemia are chromosomal abnormalities, ionizing radiation, occupational exposure to benzene, hair dyes, viruses, diet [27], [29], [44], [46], [49], [50]. The International Agency for Research on Cancer expert working group confirmed the association between tobacco smoking and myeloid leukemia [51]. The tobacco exposure in Serbia is high. In 2000, 48% of men and 38% of women in Serbia were smokers [47]. One case control study suggested that MTHFR 677 gene variants have no significant influence on the susceptibility to chronic myeloid leukemia in the Serbian population [52]. An earlier case control study has shown that working in a hazardous industry, hair dye use, family history of leukemia, exposure to electromagnetic radiation and brick mortar exposure was significantly related to chronic lymphocytic leukemia in Serbia [53]. In the Bega canal, which is one among many heavily polluted canals in Vojvodina (the province of Serbia), it was found that sediment is contaminated with (238)U and (137)Cs [54]. The origin of this contamination is discussed.

The persistent favourable trends for all age-specific mortality rates from Hodgkin's lymphoma are recorded in the observed period in Serbia. Contrary, in some populations [55] the increasing age trends in Hodgkin's disease mortality rates showed steep increase trend in 15–30 years group and a deflection at age 30 and over, which could be attributed to either progressive improvements in diagnosis and certification or to increasing exposures to risk factors. The steep upward trends in non-Hodgkin's lymphoma, multiple myeloma, lymphoid leukemia and myeloid leukemia mortality in elderly in Serbia are not fully understood, since it may be due to better diagnosis and death certification in the elderly and/or a real increase in incidence, with the most likely explanation being an increase in exposure to risk factors.

The last decades have several major improvements in the treatment of leukemias and lymphomas in Serbia, such as introduction of bone marrow transplantation (available in two hospitals), imatinib in Serbia was authorized by Medicines and Medical Devices Agency of Serbia in 2002, and rituximab in 1998, and the standard treatment for each MLHN disease in Serbia was approved according to the world standard recommendations [56], [57], [58]. In Serbia, which is one of the medium-developed countries on the basis of the gross domestic income, expenditures for health care per capita are much lower than in well-developed countries, and therefore the availability of treatment has not always been gratifying in the past.

### Strengths and limitations of the study

The present study is the only study that has reported data on the mortality trends from MLHN in Serbia in the last two decades. This study highlights the necessity for widespread adoption of the contemporary treatment for MLHN in Serbia, which may lead to improved outcomes and the avoidance of a substantial number of deaths. Also, this study provides a basis for comparison across regions. These trends are crucial not only for monitoring of the epidemiological situation regarding MLHN, but also for evaluation of various preventive and therapeutic measures.

The question about the validity of causes of death reported in national statistics was a limitation. World Health Organization assessed data quality and confirmed that Serbia has comprehensive death registration systems and judged that cause-of-death data were of moderate quality: the proportion of cases with uncertain death cause was <10% [40]. Advantages of mortality statistics data in Serbia are national coverage and the consistent quality. Registration data was 100% complete for the population of Serbia, with death registration coverage of 97% [40]. Based on the World Health Organization's latest estimates, data for the most recent years concerning percentages of ill-defined causes of deaths and ill-defined cancer deaths in Serbia suggest further improvement in the quality of mortality data (4.4 and 3.8, respectively) [59]. The proportion of cases with uncertain death cause (revision 9 codes 780–799 and revision 10 codes R00–R99) in the observed period was on an average 6.8%, with a non-significant decreasing trend (*P* = 0.137), so that a significant increase in mortality from MLHN could hardly be attributed only to the improvement of the quality of mortality statistics in Serbia. Although the increased mortality trends from the MLHN (in overall and by types: non-Hodgkin's lymphoma, multiple myeloma and myeloid leukemia) are real, a limitation of this study could be related to the potential impact of the cases which were registered as Lymphoma or Leukemia not otherwise specified (NOS). So far, the number of deaths for the particular lymphoma and leukemia subtypes in Serbia was not available. Namely, the Statistical Office of the Republic of Serbia plans to control the fourth characters in the database of deceased starting from 2015, according to Eurostat recommendations. It is important to continue efforts to assess and improve the quality of the mortality statistics. Although changes and improvements in the classification and coding of lymphomas and leukemia may have had some influence on the mortality trends, the reasons for the observed increased mortality are still insufficiently understood.

Absence of reliable data on incidence of MLHN, therapy and survival in Serbia during the observed period was the study's limitation. Serbia experienced substantial social and economic changes during the studied period, which could affect distribution of risk factors and the quality of medical care. The lack of data on changes in risk factors for MLHN in Serbia made it impossible to adjust mortality rates for these factors and to directly examine their impact on changes in the rates.

This study could be an important resource for epidemiological and other types of studies in the future.
